# Covid Pandemic Effects on the Physical Fitness of Primary School Children: Results of the German EMOTIKON Project

**DOI:** 10.1186/s40798-023-00624-1

**Published:** 2023-08-14

**Authors:** Paula Teich, Thea Fühner, Florian Bähr, Christian Puta, Urs Granacher, Reinhold Kliegl

**Affiliations:** 1 Division of Training and Movement Science, Faculty of Human Sciences, University of Potsdam, Potsdam, Germany; 2 Division of Sports and Movement Sciences, Faculty of Educational Sciences, University of Erfurt, Erfurt, Germany; 3 Department of Sports Medicine and Health Promotion, Friedrich-Schiller-University of Jena, Jena, Germany; 4 Center for Interdisciplinary Prevention of Diseases Related to Professional Activities, Jena, Germany; 5 Center for Sepsis Control and Care (CSCC), Jena University Hospital/ Friedrich-Schiller-University Jena, Jena, Germany; 6 Department of Sport and Sport Science, Exercise and Human Movement Science, University of Freiburg, Freiburg, Germany

**Keywords:** Sars-CoV-2, Cohort study, Cardiorespiratory endurance, Muscle power, Physical fitness, Youth, EMOTIKON, Linear mixed models

## Abstract

**Background:**

In spring of 2020, the Sars-CoV-2 incidence rate increased rapidly in Germany and around the world. Throughout the next 2 years, schools were temporarily closed and social distancing measures were put in place to slow the spread of the Covid-19 virus. Did these social restrictions and temporary school lockdowns affect children’s physical fitness? The EMOTIKON project annually tests the physical fitness of all third-graders in the Federal State of Brandenburg, Germany. The tests assess cardiorespiratory endurance (6-min-run test), coordination (star-run test), speed (20-m sprint test), lower (powerLOW, standing long jump test), and upper (powerUP, ball-push test) limbs muscle power, and static balance (one-legged stance test with eyes closed). A total of 125,893 children were tested in the falls from 2016 to 2022. Primary analyses focused on 98,510 keyage third-graders (i.e., school enrollment according to the legal key date, aged 8 to 9 years) from 515 schools. Secondary analyses included 27,383 older-than-keyage third-graders (i.e., OTK, delayed school enrollment or repetition of a grade, aged 9 to 10 years), who have been shown to exhibit lower physical fitness than expected for their age. Linear mixed models fitted pre-pandemic quadratic secular trends, and took into account differences between children and schools.

**Results:**

Third-graders exhibited lower cardiorespiratory endurance, coordination, speed and powerUP in the Covid pandemic cohorts (2020–2022) compared to the pre-pandemic cohorts (2016–2019). Children’s powerLOW and static balance were higher in the pandemic cohorts compared to the pre-pandemic cohorts. From 2020 to 2021, coordination, powerLOW and powerUP further declined. Evidence for some post-pandemic physical fitness catch-up was restricted to powerUP. Cohen’s |*d*s| for comparisons of the pandemic cohorts 2020–2022 with pre-pandemic cohorts 2016–2019 ranged from 0.02 for powerLOW to 0.15 for coordination. Within the pandemic cohorts, keyage children exhibited developmental losses ranging from approximately 1 month for speed to 5 months for cardiorespiratory endurance. For powerLOW and static balance, the positive pandemic effects translate to developmental gains of 1 and 7 months, respectively. Pre-pandemic secular trends may account for some of the observed differences between pandemic and pre-pandemic cohorts, especially in powerLOW, powerUP and static balance. The pandemic further increased developmental delays of OTK children in cardiorespiratory endurance, powerUP and balance.

**Conclusions:**

The Covid-19 pandemic was associated with declines in several physical fitness components in German third-graders. Pandemic effects are still visible in 2022. Health-related interventions should specifically target those physical fitness components that were negatively affected by the pandemic (cardiorespiratory endurance, coordination, speed).

**Supplementary Information:**

The online version contains supplementary material available at 10.1186/s40798-023-00624-1.

## Key points

- Third-graders exhibited lower cardiorespiratory endurance, coordination, speed, and upper limbs muscle power in the Covid cohorts (2020–2022) compared to the pre-pandemic cohorts (2016–2019). Children’s lower limbs muscle power and static balance were higher in the pandemic compared to the pre-pandemic cohorts.
- Pandemic-related effects on the physical fitness of children are small in effect size, but reflect developmental losses of approximately 5 months for cardiorespiratory endurance, 3 months for coordination, 1 month for speed, and 2 months for upper limbs muscle power. Developmental gains of 1 month were found for lower limbs muscle power and 7 months for static balance. However, pre-pandemic secular trends explain some of the observed changes, especially in lower and upper limbs muscle power, and static balance.
- Children with delayed school enrollment already exhibited poorer physical fitness than expected for their age before the pandemic. Their physical fitness impairments further increased during the pandemic years.

**Supplementary Information:**

The online version contains supplementary material available at 10.1186/s40798-023-00624-1.

## Background

In spring of 2020, the Sars-CoV-2 incidence rate increased rapidly in Germany and around the world [1]. Throughout the next two years, schools were temporarily closed and contact restrictions were put in place to slow the spread of the Covid-19 virus. For some periods, children were locked out of sports in schools and sports clubs, which often resulted in decreased physical activity [2]. Did these restrictions affect children’s physical fitness? And if so, has children’ s physical fitness improved after the pandemic?

In youth, physical fitness is an important influencing factor of their current [3, 4] and a predictor for their future [5, 6] health. Cardiorespiratory endurance and muscular fitness are negatively associated with cardiometabolic risk factors [7], such as estimates of body fat [3, 4] or insulin resistance [7]. The role of children’s physical fitness extends to psychological outcomes, with studies reporting positive associations with health-related quality of life [8], executive function [9, 10], and academic achievement [11, 12].

Various studies from around the world reported decreased physical activity levels [13–16], and increased sedentary behavior [13, 15] in children and adolescents during the Covid-19 pandemic. Findings from a German study showed an increase in physical activity levels, mainly driven by increased habitual physical activity, but a decrease in sports activities during the first lockdown in spring 2020 [17]. The increase in habitual physical activity levels, however, did not last until the second lockdown at the start of 2021 [18]. It is possible that the limited opportunities to perform structured exercise in physical education classes or sports clubs due to social-distancing measures negatively affected children’s physical fitness. Several studies reported declines in children’s cardiorespiratory endurance and changes in their muscular fitness [19–26], although results regarding muscular fitness were inconsistent [19–22, 25–28].

For instance, Austrian researchers tested the physical fitness of 24,571 primary school children either before (i.e., between 2016 and 2019) or after the Covid-19 pandemic (i.e., in 2022). Performance in the 6-min run test and in an agility-run test was lower in 2022 compared to the pre-pandemic cohorts, while performance in the medicine ball push test was better after the pandemic [21]. In Germany, Wessely et al. [25] tested the physical fitness of 1238 eight-year-old children in the falls of 2016, 2020, or 2021. Social burden was determined by a school index based on socioeconomic indicators like migration and parental income status. From 2016 to 2021, performance in the 6-min run test declined, with a larger decline for children from schools with a high social burden. The authors further reported that standing long jump performance increased from 2016 to 2020 in children with a high social burden. Another German research group tested 999 primary school children between 2012 and 2021. They found no evidence of pandemic-related declines in children’s cardiorespiratory endurance, but also reported a better standing long jump test performance in the first pandemic year compared to pre-pandemic cohorts [22]. In contrast, French researchers reported that performance in the standing long jump test, medicine ball push test, and 20-m shuttle run test decreased in third- and fourth-graders during the first pandemic year [19].

When determining Covid pandemic effects on physical fitness, it is important to dissociate pandemic effects from secular trends of physical fitness. Secular trends differ between different physical fitness components [29–31]. For instance, in the years leading up to the Covid pandemic, cardiorespiratory endurance of German third-graders declined, whereas speed increased over time [32]. To further examine potential Covid-19 pandemic effects on children’s physical fitness, as well as its post-pandemic development, we report an assessment that (1) is based on large representative samples of German third-graders tested in the cohorts 2016 to 2022 (population-based assessment), and (2) takes into account pre-pandemic secular trends associated with different physical fitness components. This allows us to check whether, in principle, pre-pandemic cohort trends could account for or also underestimate pandemic-related physical fitness changes.

Because previous research has shown that physical fitness development of children differs depending on their timing of school enrollment [33, 34], we assess the Covid-19 pandemic effects on physical fitness in two different groups of children. Our primary analyses focus on *keyage children*, who were enrolled in school according to the legal key date. For the Federal State of Brandenburg, Germany, this means that they had reached the age of six by September 30 of their respective school enrollment year and were between 8 and 9 years old in third grade. Secondary analyses focus on children whose school enrollment had been delayed or who repeated a grade (i.e., *older-than-keyage children, OTK*); they were between 9 and 10 years in third grade. In contrast to keyage children, whose physical fitness development is linear between the ages of eight and nine [32], OTK children fall short of the physical fitness expected for their age, with larger deviations between predicted and observed performance for relatively older OTK children [33]. We test whether the Covid pandemic further exacerbated the physical fitness deficits of OTK children.

## Methods

### Experimental Approach

The EMOTIKON project [35] annually assesses the physical fitness of all third-graders in the Federal State of Brandenburg, Germany. EMOTIKON was mandated and approved by the Ministry of Education, Youth and Sport of the Federal State of Brandenburg [36]. According to the Brandenburg School Law, participation is mandatory for public primary schools. The physical fitness tests were conducted between August and December. Prior to testing, schools and parents received written information about the EMOTIKON study, including instructions on test administration and information on data processing and data protection. The authors received the data completely anonymized from the Ministry of Education, Youth and Sport of the Federal State of Brandenburg. Research was conducted in accordance with the latest Declaration of Helsinki [37] and the Brandenburg School Law [36].

This report builds on Fühner et al. [32, 33] with new data for the cohorts 2020, 2021, and 2022 added to data from the pre-pandemic cohorts 2016 to 2019. Obviously, given the overlap (Fühner et al. analyzed age and sex effects on physical fitness using data of children tested between 2011 and 2019), we do not expect much of a difference as far as age and sex effects are concerned; these effects assess the stability of results reported previously. Restricting analyses to data from cohorts 2016 until 2022 allowed us to include an indicator of a sixth physical fitness component, that is static balance. This test has been available only since 2016, replacing a flexibility test used between 2011 and 2015 [38]. Finally, as the focus is on the Covid pandemic effect and as there were particularly pronounced cohort effects relating to the 2011 to 2015 cohorts, the selection used here gives more weight to recent secular trends. Analyses using all data from 2011 to 2022 are reported in the Supplement and in the Open Science Framework (OSF) repository https://osf.io/w975d/ [39]. Following Fühner et al. [32], the primary analyses are restricted to keyage children. In a second set of analyses, we test whether the Covid pandemic increased the OTK children’s deviations between observed and predicted physical fitness reported previously [33, 34].

### Population

Overall, 125,893 children from seven cohorts (2016 until 2022) and 515 schools in the Federal State of Brandenburg, Germany, were included in the analyses. Of these children, 98,510 were keyage third-graders (i.e., school enrollment according to the legal key date, between 8 and 9 years in third grade). In addition, data from 27,383 OTK children (i.e., delayed school enrollment or repetition of a school grade, between 9 and 10 years in third grade) in 514 schools were included in analyses. Tables 1 and 2 provide an overview of the sample characteristics of keyage and OTK children, respectively. Additional file 1: Tables S1 and S2 provide detailed information about means and standard deviations for all test scores before, during, and after the pandemic for keyage and OTK children.

**Table 1:** Sample description of keyage children before (i.e., cohorts 2016–2019) and during or after (i.e., cohorts 2020–2022) the Covid-19 pandemic

Cohorts	N children (% girls)	N test scores	Age [years] M (SD)	N schools
Pre-pandemic (2016–2019)	54,343 (51%)	315,260	8.62 (0.28)	500
During and after the pandemic (2020–2022)	44,167 (51%)	255,526	8.56 (0.28)	495
Total	98,510 (51%)	570,786	8.59 (0.28)	515

**Table 2:** Sample description of OTK children before (i.e., cohorts 2016–2019) and during or after (i.e., cohorts 2020–2022) the Covid-19 pandemic

Cohorts	N children (% girls)	N test scores	Age [years] M (SD)	N schools
Pre-pandemic (2016–2019)	14,210 (41%)	82,150	9.38 (0.25)	499
During and after the pandemic (2020–2022)	13,173 (42%)	75,601	9.34 (0.27)	495
Total	27,383 (42%)	157,751	9.36 (0.26)	514

### Physical Fitness Tests

The EMOTIKON test battery comprises six physical fitness tests. The 6-min run test assesses cardiorespiratory endurance, the star-run test assesses coordination, the 20-m linear sprint tests speed, the standing long jump test and the ball-push test assess proxies of lower and upper limbs muscle power, and the one-legged stance test with eyes closed tests static balance. The physical fitness tests were administered by physical education teachers, following a standardized procedure (for more details, see the project’s website [35]). Before test administration, children received warm-up exercises consisting of running exercises or games (e.g., playing tag). Children were encouraged to achieve their best performance in the physical fitness tests.

#### Cardiorespiratory Endurance

The 6-min run test assessed children’s cardiorespiratory endurance. For 6 min, the children ran as far as they could around a volleyball field measuring 9 m × 18 m = 54 m. The field was marked using pylons that were set at a 9 m distance from each other. If a child stopped between two pylons at the stop signal, they were allowed to continue to the next pylon. The total distance covered during the six minutes up to that last pylon was recorded in meters. In children aged 7 to 11 years, the 6-min run test showed a high test–retest reliability of *r* = 0.92 [40].

#### Coordination

The star-run test assessed coordination under time pressure. Children had to run a star-like pattern with the total distance of 50.912 m as fast as possible. Four pylons marked the corners of a 9 m × 9 m square, and one pylon marked the midpoint. Starting from the midpoint, children had to run to each of the other four pylons, touch it by hand and run back to the midpoint. During this task, they had to use different movement directions and movement forms (i.e., running forward, running backward, side-steps to the right side, side-steps to the left side) in a standardized order. The children had two trials. Time was measured in seconds with a 1/10 s accuracy. The score of the fastest trial was used in the analysis. The star-run test showed a test–retest reliability (intra-class correlation coefficient, ICC) of 0.68 (95% CI 0.53–0.79) in children between 8 and 10 years [41].

#### Speed

The 20-m linear sprint test assessed the physical fitness component speed. The children started the sprint from a standing position after an acoustic signal. Time was measured in seconds with a 1/10 s accuracy. Children had two trials. The faster of the trials was used in the analysis. The 20-m sprint test showed a test–retest reliability of *r* = 0.90 in children between 7 and 11 years [40].

#### Lower Limbs Muscle Power (PowerLOW)

The standing long jump test has frequently been applied as a proxy to estimate lower limbs muscle power (powerLOW). Children had to jump as far as possible out of a standing position with their feet parallel and shoulder-wide. They had to jump with both legs concurrently and land with both feet together. Children were allowed to swing their arms before and during the jump, but they were not allowed to touch the floor with their hands after landing. The distance between their toes at take-off and their heels at landing (or the heel of the rear foot, if their feet were not parallel at landing) was measured in centimeters with a 1 cm accuracy. The children had two trials. The best trial was used in the analysis. The standing long jump test showed a test–retest reliability (ICC) of 0.94 (95% CI 0.93–0.95) in children between 6 and 12 years [42].

#### Upper Limbs Muscle Power (PowerUP)

The ball-push test is a proxy to assess upper limbs muscle power (powerUP). Children stood in an upright position with their feet shoulder-width apart. They held a 1 kg medicine ball in front of their chest. The children’s task was to push the ball with both hands as far as possible in horizontal direction. The distance was measured in meters with a 10 cm accuracy. Again, the children had two trials. The trial with the best result was used in analysis. The ball-push test showed a test–retest reliability (ICC) of 0.81 (95% CI 0.71–0.87) in children between 8 and 10 years [41].

#### Static Balance

The one-legged stance test with eyes closed assessed children’s static balance. The children stood with hands held akimbo, their standing leg slightly bent, both knees pointing forward, and the free leg bent between 60° and 90° at the hip joint and approximately 90° at the knee joint. This position was visually controlled by the physical education teacher. After they were in this position, they closed their eyes, the test started and participants remained in this quiet position for as long as possible. The maximum duration of a test trial was 60 s, after which the test was terminated. Time was measured in seconds with a 1 s accuracy. Only if the children’s test trial lasted less than five seconds, they were granted another trial. For scores higher than 60 s, indicating that the individual showed optimal test performance, test time was set at a maximal value of 60 s. The one-legged stance test with eyes closed showed a test–retest reliability (ICC) of 0.69 (95% CI 0.61–0.75) in children between 7 and 10 years [43].

### Statistics

We preprocessed and analyzed data with *R* (4.2.3) [44], the *RStudio IDE* [45], *Julia* (Version 1.9.0) [46], and *VS Code IDE* [47]. For data preprocessing we used *tidyverse* [48] and *easystats* [49] suites of packages. Linear mixed models (LMMs) were estimated with the *MixedModels.jl* package [50] in *Julia*. We used the *JellyMe4.jl* package [51] and the *MixedModelsExtras.jl* package [52] for data analysis and postprocessing of LMMs. Details regarding parsimonious model selection [53] are documented in analysis scripts in the OSF repository. The Covid-19 pandemic effects on physical fitness of keyage and OTK children were tested with separate LMMs for each group of children.

Consistent with previous reports [32–34], a box-cox distributional analysis [54] indicated that for the star-run test and the 20-m sprint test, a reciprocal transformation, and for the one-legged stance test, a logarithmic transformation of the test scores was required for a normal distribution of model residuals. The original unit of the star-run and the 20-m sprint test was seconds. We transformed their units into meter/second by multiplying the reciprocal scores (1/s) of the star-run with 50.912 (distance in meters of the star-run) and the reciprocal scores of the 20-m sprint with 20 (distance in meters of the 20-m sprint). Consequently, higher scores indicate better performances for all six physical fitness tests.

### Analysis of the Covid-19 Pandemic Effects on the Physical Fitness of Keyage Children

The analysis started with 98,521 keyage children from 515 schools in the cohorts from 2016 until 2022. We excluded three children for whom information on their gender was not provided. Based on teachers’ notes, we excluded five children with a physical disability and one child with an autism diagnosis. To identify outliers, we calculated z-scores separately for boys and girls for each test. For all tests, except for the one-legged stance test, we excluded scores outside of a ± 3 *SD* range (i.e., 1646 test scores [0.3%] were excluded). As the one-legged-stance test was terminated after 60 s of successful performance and scores larger than 60 s were not possible, the whole test score range indicates valid performance. We thus did not apply the ± 3 *SD* criterion for this test. This left us with 570,786 test scores from 98,510 children in 515 schools. Finally, z-scores were recalculated separately for each test, aggregated over boys and girls, to keep sex-related differences in the dependent variable.

#### Linear Mixed Model for Keyage Children

The six physical fitness components were treated as six factor levels of the factor ‘physical fitness component’. For this factor, we specified five contrasts comparing (1) cardiorespiratory **e**ndurance, **c**oordination, and **s**peed versus power**L**OW, power**U**P, and static **b**alance (i.e., three running tests against tests assessing muscular power and balance, ECS vs. LUB), (2) cardiorespiratory **e**ndurance and **c**oordination versus **s**peed (EC vs. S), (3) cardiorespiratory **e**ndurance versus **c**oordination (E vs. C), (4) power**L**OW versus power**U**P (L vs. U), and (5) power**U**P versus static **b**alance (U vs. B). For the seven-level factor cohort (i.e., 2016–2022), five indicator variables tested (1) the physical fitness difference between the pre-pandemic cohorts (i.e., 2016–2019) and the cohorts tested since the start of the pandemic (2020–2022), (2) the physical fitness difference between the first and second pandemic year (i.e., 2020 vs. 2021), and (3) the physical fitness difference between cohorts 2021 and 2022 (i.e., a possible ‘rebound’ effect after the Covid-19 pandemic). Finally, two orthogonal polynomial contrasts testing (4) linear and (5) quadratic pre-pandemic secular trends informed about potential confounds of the overall pandemic effect due to cohort-related changes. The factor ‘sex’ contrasted boys and girls with positive scores indicating better performance for boys. Age was centered at 8.5 years.

Parsimonious model selection (i.e., an LMM with variance components [VCs] and correlation parameters [CPs] supported by the data and pruning of high-order interaction of fixed effects unless motivated for theoretical reasons) started with a model including fixed effects of sex, age, and cohort, as well as interactions between sex and the five cohort indicator variables, all nested under the six levels of the factor physical fitness component. We reduced the complexity of the fixed-effect structure by excluding interactions between sex and the cohort indicator variables without loss in goodness of model fit. Random factors were child and school. For both random factors, we included VCs and CPs for the contrasts defined for the six physical fitness components. The random factor school also included age-, sex-, and cohort-related VCs and age- and sex-effect related CPs. Details about parsimonious model selection are documented in script *keyage_lmm_16_22.qmd* in the OSF repository. We interpreted fixed effects with |z-values| > 2 as significant.

### Analysis of Covid-19 Pandemic Effects on the Physical Fitness of Older-than-Keyage Children

OTK children exhibit lower physical fitness than expected for their age [33, 34]. Therefore, we tested whether the Covid pandemic further increased or reduced differences between OTK children’s expected and observed physical fitness. We started out with 30,283 OTK children from 514 schools in the cohorts from 2016 until 2022. We excluded children older than 10 years (i.e., 2896 children were excluded). Based on teachers’ notes, we further excluded two children with a physical disability and one child with autism spectrum disorder. We only kept children from the same schools as keyage children, leaving us with 27,384 OTK children.

Computation of z-scores was adopted from Fühner et al. [33] and was done in two steps. First, we calculated z-scores separately for each test (i.e., 6-min-run test, star-run test, 20-m linear sprint test, standing long jump test, ball-push test and one-legged stance test) x sex (boy, girl) cell. For all tests, except for the one-legged stance test, we excluded scores outside of a ± 3 *SD* range (387 test scores [0.2%] excluded). This left us with 157,751 test scores from 27,383 OTK children in 514 schools. In a second step, z-scores were recalculated separately for each test (aggregated over boys and girls to keep sex-related differences in the data) using means and *SD*s from 98,510 *keyage* children from the same cohorts. As Fühner et al. [33], we predicted test performance for OTK children based on the LMM for keyage children reported in the present study; as for keyage children, age was centered at 8.5 years. The difference between observed (i.e., z-scores computed from physical fitness test scores) and predicted performance (i.e., z-scores predicted based on LMM from keyage children) is expressed in *delta z-scores* (i.e., observed z-scores–predicted z-scores). *Delta z-scores* indicate that the observed test performance fell short of the predicted performance (i.e., negative *delta z-score*) or was higher than predicted (i.e., positive *delta z-scores*).

#### Linear Mixed Model for OTK Children

In general, the LMM for OTK children’s *delta z-scores* was expected to be less complex than the LMM for keyage children because fixed effects related to contrasts of physical fitness component, cohort (i.e., five indicator variables testing pandemic effects as well as pre-pandemic secular trends), age (linear), and sex as well as school-related random effects were already part of the predicted z-scores. Moreover, the smaller number of children implied also lower statistical power. Details regarding parsimonious model selection are reported in script *otk_lmm_delta_16_22.qmd* in the OSF repository. In contrast to the keyage LMM, we included a two-level Covid factor comparing the pre-pandemic cohorts (i.e., 2016–2019) with the cohorts during or after the pandemic (i.e., 2020–2022).

For the factor ‘physical fitness component’, we used the same contrast coding as for keyage children. The final LMM included fixed effects Covid, sex, and age (i.e., a second-order polynomial trend), all nested under the six levels of physical fitness component. A significant negative Covid pandemic effect for OTK children indicates that OTK children’s physical fitness deficits were exacerbated relative to the physical fitness of keyage children. Random effects were child and school. For the random factor child, we included physical fitness component related VCs and CPs. For the random factor school, we included physical fitness component, Covid, and age (linear) related VCs.

## Results

### Linear Mixed Model for Keyage Children

**Fig. 1:**
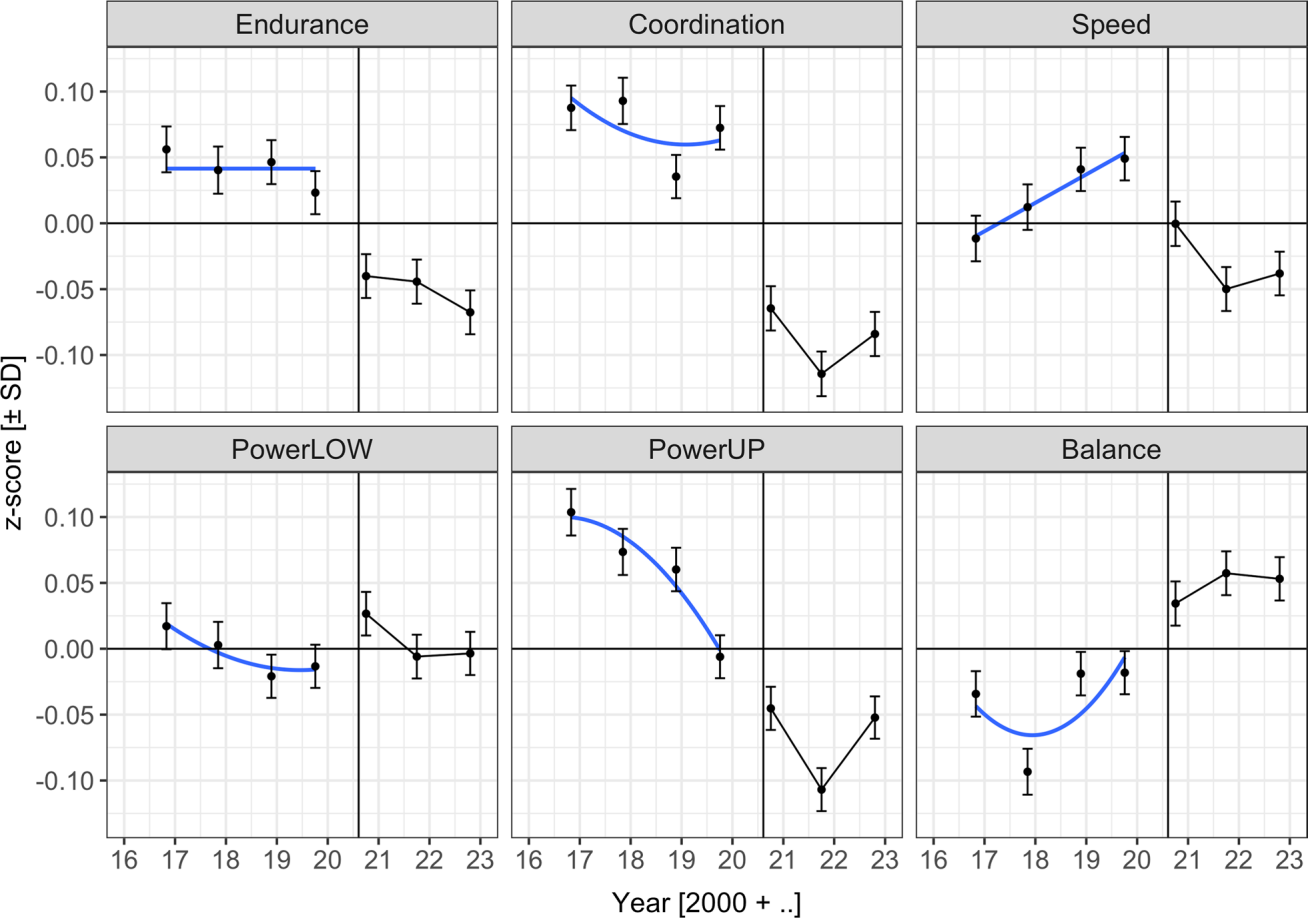
Mean z-scores and 95% CIs for the cohorts 2016 to 2022 for six physical fitness components. The vertical line marks the first day of the school year in which the first Covid cohort was tested (August 10, 2020). For Coordination, PowerLOW, PowerUP, and Balance, the blue lines show a quadratic pre-pandemic cohort trend. For Speed, the blue line shows a linear pre-pandemic cohort trend. For Endurance neither the linear, nor the quadratic cohort trend were significant; the blue line therefore marks the pre-pandemic cohort mean. Endurance = cardiorespiratory endurance (i.e., 6-min-run test), Coordination = star-run test, Speed = 20-m linear sprint test, PowerLOW = lower limbs muscle power (i.e., standing long jump test), PowerUP = upper limbs muscle power (i.e., ball-push test), balance = static balance (i.e., one-legged-stance test with eyes closed). For coordination and speed, scores were converted from seconds to meter/second (i.e., pace scores; star-run test = 50.912 [m]/time [s]; 20-m linear sprint test = 20 [m]/time [s]). For static balance, scores were log-transformed

Figure 1 displays keyage children’s performance profiles for the six physical fitness components for the 2016 to 2022 cohorts. Mean z-scores for cohorts are depicted as black points. Pre-pandemic secular trends are shown in blue. The vertical line marks the first day of the school year in which the first Covid cohort was tested (August 10, 2020). Cohort means are shown at the mean test date for each cohort. Table 3 shows fixed-effect LMM estimates, standard errors, and z-values of the corresponding LMM.

**Table 3:** Fixed effect estimates, standard errors, and z-values of the LMM for keyage children

Source of variance	Fixed-effect estimate	Standard error	z-value
Grand mean (intercept)	− 0.044	0.010	− 4.64
Physical fitness component			
ECS versus LUB	0.028	0.011	**2.51**
EC versus S	− 0.005	0.016	− 0.34
E versus C	0.011	0.019	0.56
L versus U	0.006	0.015	0.43
U versus B	0.045	0.020	**2.28**
*Cohort contrasts*			
Endurance (6-min run test)			
Pre1 (linear)	− 0.029	0.061	− 0.48
Pre2 (quadratic)	0.014	0.025	0.56
Covid contrast 1	− 0.077	0.011	− **7.32**
Covid contrast 2	− 0.022	0.016	− 1.35
Covid contrast 3	− 0.011	0.016	− 0.65
Coordination (star-run test)			
Pre1 (linear)	− 0.060	0.061	− 0.98
Pre2 (quadratic)	0.093	0.025	**3.72**
Covid contrast 1	− 0.146	0.011	− **13.78**
Covid contrast 2	− 0.037	0.016	− **2.29**
Covid contrast 3	0.025	0.016	1.55
Speed (20-m sprint test)			
Pre1 (linear)	0.230	0.061	**3.76**
Pre2 (quadratic)	0.031	0.025	1.26
Covid contrast 1	− 0.029	0.011	− **2.78**
Covid contrast 2	− 0.030	0.016	− 1.87
Covid contrast 3	0.013	0.016	0.82
PowerLOW (standing long jump test)			
Pre1 (linear)	− 0.085	0.062	− 1.38
Pre2 (quadratic)	0.070	0.025	**2.78**
Covid contrast 1	0.022	0.011	**2.06**
Covid contrast 2	− 0.039	0.016	− **2.39**
Covid contrast 3	0.006	0.016	0.38
PowerUP (ball-push test)			
Pre1 (linear)	− 0.241	0.060	− **4.00**
Pre2 (quadratic)	0.064	0.025	**2.62**
Covid contrast 1	− 0.083	0.010	− **7.96**
Covid contrast 2	− 0.065	0.016	− **4.07**
Covid contrast 3	0.044	0.016	**2.78**
Balance (one-legged-stance test)			
Pre1 (linear)	0.031	0.061	0.51
Pre2 (quadratic)	0.068	0.025	**2.71**
Covid contrast 1	0.080	0.011	**7.58**
Covid contrast 2	0.024	0.016	1.47
Covid contrast 3	0.003	0.016	0.18
Age (linear) nested within physical fitness component			
Endurance: a1	0.072	0.011	**6.55**
Coordination: a1	0.274	0.011	**24.96**
Speed: a1	0.202	0.011	**18.32**
PowerLOW: a1	0.209	0.011	**18.56**
PowerUP: a1	0.516	0.011	**48.98**
Balance: a1	0.132	0.011	**12.09**
Sex nested within physical fitness component			
Endurance: Sex	0.435	0.006	**69.29**
Coordination: Sex	0.225	0.006	**35.64**
Speed: Sex	0.296	0.006	**46.81**
PowerLOW: Sex	0.380	0.007	**58.76**
PowerUP: Sex	0.663	0.006	**109.22**
Balance: Sex	− 0.244	0.006	**-38.98**

Physical fitness contrasts: ECS versus LUB = endurance, coordination, and speed versus powerLOW, powerUP, and balance. EC versus S = endurance and coordination versus speed. E versus C = endurance versus coordination. L versus U = powerLOW versus powerUP. U versus B = powerUP versus balance. Cohort contrasts: Pre1 (linear) = Linear pre-pandemic secular trend. Pre2 (quadratic) = Quadratic pre-pandemic secular trend. Covid contrast 1 = Cohorts 2016–2019 versus 2020–2022. Covid contrast 2 = Cohort 2020 versus 2021. Covid contrast 3 = Cohort 2021 versus 2022. Endurance = cardiorespiratory endurance (i.e., 6-min run test), coordination = star-run test, speed = 20-m linear sprint test, powerLOW = lower limbs muscle power (i.e., standing long jump test), powerUP = upper limbs muscle power (i.e., ball-push test), Balance = static balance (i.e., one-legged-stance test with eyes closed). Bold = |z| > 2.0, linear mixed model random factors: schools (515) and children (98,510), observations = 570,786. For estimates of variance components and correlation parameters, see Table 4

#### Cohort-Related Changes in Cardiorespiratory Endurance

Performance in the 6-min run test was significantly lower in the cohorts 2020–2022 compared to the pre-pandemic cohorts (*b* = − 0.077, z = − 7.32). There was no evidence for pre-pandemic secular trends between 2016 and 2019 (|z|< 1). There was also no evidence for performance differences between the first and second pandemic year or for a post-pandemic ‘rebound’ effect (|z|< 2).

#### Cohort-related changes in Coordination

Star-run test performance was significantly lower in cohorts 2020–2022 compared to the pre-pandemic cohorts (*b* = − 0.146, z = − 13.78). Performance in the second pandemic year 2021 was lower compared to the first pandemic year (*b* = − 0.037, z = − 2.29). There was no evidence for differences between cohorts 2021 and 2022 (z < 2). Finally, there was a significant pre-pandemic positive quadratic cohort trend (*b* = 0.093, z = 3.72).

#### Cohort-Related Changes in Speed

Children’s performance in the 20-m sprint test was significantly lower in cohorts 2020–2022 compared to the pre-pandemic cohorts (*b* = − 0.029, z = − 2.78). There was no evidence for a difference between the first and second pandemic year, or for a post-pandemic rebound effect (|z|< 2). Performance increased linearly in pre-pandemic years 2016 to 2019 (*b* = 0.230, z = 3.76).

#### Cohort-Related Changes in PowerLOW

Standing long jump test performance was better in the pandemic cohorts (2020–2022) compared to the pre-pandemic cohorts (*b* = 0.022, z = 2.06). However, after the first pandemic year 2020, standing long jump performance declined (*b* = − 0.039, z = − 2.39). There was no evidence for a performance difference between cohorts 2021 and 2022 (z < 1). Pre-pandemic performance was characterized by a positive quadratic cohort trend between 2016 and 2019 (*b* = 0.070, z = 2.78).

#### Cohort-Related Changes in PowerUP

Children in cohorts 2020–2022 exhibited lower ball-push test performance compared to children in pre-pandemic cohorts (*b* = − 0.083, z = − 7.96). Performance was lower in 2021 compared to 2020 (*b* = − 0.065, z = − 4.07). After the pandemic, ball-push test performance improved, with children in 2022 exhibiting better performance compared to children in the previous cohort (*b* = 0.044, z = 2.78). Pre-pandemic performance in the ball-push test was characterized by a linear decline (*b* = − 0.241, z = − 4.00) and a positive quadratic trend (*b* = 0.064, z = 2.62).

#### Cohort-Related Changes in Static Balance

One-legged stance test performance was better after the start of the pandemic compared to the pre-pandemic cohorts (*b* = 0.080, z = 7.58). There was no evidence for performance differences between cohorts 2020 and 2021, or for a difference between the 2021 and 2022 cohorts (z < 2). Between 2016 and 2019, performance in the one-legged stance test was characterized by a positive quadratic cohort trend (*b* = 0.068, z = 2.71).

#### Age and Sex Effects on Physical Fitness

Age and sex effects on the first five physical fitness tests were in agreement with results reported by Fühner et al. [32]. For all physical fitness tests, performance increased with age. The age gain was smallest for the 6-min run test (*b* = 0.072, z = 6.55) and largest for the ball-push test (*b* = 0.516, z = 48.98). Boys outperformed girls in the 6-min run, the star-run, the 20-m sprint, the standing long jump, and the ball-push test (*b*s between 0.225 for the star-run test and 0.663 for the ball-push test, z-values between 35.64 for the star-run test and 109.22 for the ball-push test). In the present study, we also report a significant positive age effect (*b* = 0.132, z = 12.09) and a reverse sex effect for the one-legged stance test, with girls significantly outperforming boys (*b* = − 0.244, z = − 38.98).

Table 4 shows the VCs of the random effect structure and their associated CPs (i.e., correlations after all other effects in the LMM are taken into account). VCs related to the physical fitness component contrasts were larger (VCs between 0.209 and 0.884) for children than for schools (VCs between 0.059 and 0.184). There were small school-related differences in the age (0.001) and sex effects (0.002), as well as school-related differences in the five cohort contrasts (VCs between 0.032 and 0.982). We replicated several CPs reported previously [32]. The child-related CP between the physical fitness components *powerLOW versus powerUP* (i.e., L vs. U) and the children’s Grand Mean (*r* = − 0.37) indicates that “physically fitter” children (i.e., higher Grand Mean) tend to show a higher performance in the standing long jump test relative to the ball-push test. This is evidence that powerLOW, but not so much powerUP, is an indicator for physical fitness.

**Table 4:** Child- and school-related variance components and correlation parameters of the LMM for keyage children

	VC	CP						
		Grand mean	ECS versus LUB	EC versus S	E versus C	L versus U	U versus B	Age
Child								
Grand mean (Intercept)	0.276	1.00						
ECS versus LUB	0.209	− 0.23	1.00					
EC versus S	0.252	0.13	0.09	1.00				
E versus C	0.457	0.09	0.15	0.06	1.00			
L versus U	0.499	− **0.37**	0.38	− 0.13	0.16	1.00		
U versus B	0.884	− 0.10	0.10	− 0.09	− 0.15	− 0.45	1.00	
School								
Grand mean (Intercept)	0.042	1.00						
ECS versus LUB	0.059	− 0.45	1.00					
EC versus S	0.114	− 0.11	0.12	1.00				
E versus C	0.172	0.18	− 0.03	0.04	1.00			
L versus U	0.104	− 0.23	0.12	0.07	0.00	1.00		
U versus B	0.184	0.03	0.30	− 0.07	0.13	− 0.47	1.00	
Age	0.001	**0.73**	− 0.40	− 0.05	− 0.16	− 0.15	− 0.08	
Sex	0.002	0.05	0.11	0.00	− 0.27	− 0.15	0.34	0.42
Pre1 (linear)	0.982							
Pre2 (quadratic)	0.148							
Covid contrast 1	0.032							
Covid contrast 2	0.059							
Covid contrast 3	0.062							

Physical fitness contrasts: ECS versus LUB = endurance, coordination, and speed versus powerLOW, powerUP, and balance. EC versus S = endurance and coordination versus speed. E versus C = endurance versus coordination. L versus U = powerLOW versus powerUP. U versus B = powerUP versus balance. Cohort contrasts: Pre1 (linear) = Linear pre-pandemic secular trend. Pre2 (quadratic) = Quadratic pre-pandemic secular trend. Covid contrast 1 = Cohorts 2016–2019 versus 2020–2022. Covid contrast 2 = Cohort 2020 versus 2021. Covid contrast 3 = Cohort 2021 versus 2022. Endurance = cardiorespiratory endurance (i.e., 6-min run test), Coordination  = star-run test, Speed = 20-m linear sprint test, PowerLOW = lower limbs muscle power (i.e., standing long jump test), PowerUP = upper limbs muscle power (i.e., ball-push test), balance = static balance (i.e., one-legged-stance test with eyes closed), VC = variance component, CP = correlation parameter. Theoretically relevant correlations discussed in the text are set in bold. VC for Residual = 0.322

As reported by Fühner et al. [32] and reported in Additional file 1: Table S4, in a reparameterized version of this LMM that included child-related CPs between test levels instead of between test contrasts, the four physical fitness tests assessing cardiorespiratory endurance, coordination, speed and powerLOW correlated highly with each other (mean* r*: 0.67; range 0.57 to 0.81). Thus, the four tests clearly represent the latent construct *physical fitness*. The correlations of the tests assessing powerUP and balance with the other physical fitness tests were smaller (mean *r*: 0.33; range 0.21 to 0.54); the correlation between powerUP and balance was *r* = 0.09.

The lower half of Table 4 shows school-related VCs and CPs. As reported previously [32], the schools’ Grand Mean of their children’s physical fitness correlated positively with the age effect (*r* = 0.73; bootstrapped 95% confidence interval: 0.40–0.91), indicating that physically “fitter” schools exhibited larger cross-sectional age gains in the ninth year of life. The correlation between the school’s Grand Mean and the age effect was higher in the present study than the 0.48 correlation reported by Fühner et al. [32].

### How Meaningful are the Covid-19 Pandemic Effects?

When comparing performance in the pandemic cohorts 2020–2022 with performance in the pre-pandemic cohorts 2016–2019, we found negative pandemic effects on the three run tests and on performance in the ball-push test. For the standing long jump test and the one-legged-stance test, performance in the Covid cohorts was better than in the pre-pandemic cohorts. These effects are statistically significant, but do they also have practical relevance, or are they rather small and negligeable? There are different methodological approaches for assessing the practical relevance of a significant effect. Table 5 shows the pandemic-related effects on physical fitness and their translation into four different effect-size measures.

**Table 5:** Covid-19 pandemic effects expressed in different effect size measures

	Endurance	Coordination	Speed	PowerLOW	PowerUP	Balance
Cohen’s *d* (fixed effect estimate)	− 0.08	− 0.15	− 0.03	+ 0.02	− 0.08	+ 0.08
Covid pandemic effect (2016–2019 versus 2020–2022) in test metric	− 11.7 m	− 0.038 m/s	− 0.009 m/s	+ 0.5 cm	− 0.06 m	+ 0.07 log(s)
Smallest meaningful change (SMC)	− 31.4 m	− 0.059 m/s	− 0.086 m/s	+ 3.9 cm	− 0.15 m	+ 0.18 log(s)
Developmental costs/gains during the Covid pandemic (i.e., 2020–2022) relative to 1-year development*	− 5 mth	− 3 mth	− 1 mth	+ 1 mth	− 2 mth	+ 7 mth

Covid-19 pandemic effects = Comparison of cohorts 2016–2019 with cohorts 2020–2022. As test scores were transformed to z-scores, the LMM estimates indicate changes in performance in units of *SD*. Fixed effect estimates from the LMM can thus be interpreted as Cohen’s *d*. Endurance = cardiorespiratory endurance (i.e., 6-min run test), Coordination = star-run test, Speed = 20-m linear sprint test, PowerLOW = lower limbs muscle power (i.e., standing long jump test). PowerUP = upper limbs muscle power (i.e., ball-push test), balance = static balance (i.e., one-legged-stance test with eyes closed), mth = months

*Covid pandemic effect relative to longitudinal age effect from LMMs based on test scores from 1,013 keyage children tested in third and one year later in fourth grade [34]

The first row depicts the effect sizes of the Covid pandemic effects (i.e., comparing cohorts 2020–2022 with cohorts 2016–2019). Since the dependent variable of the main LMM is a z-score, the estimates describe changes in *SD* units and can be interpreted as Cohen’s *d*s. Common effect sizes differ between different research fields, with relatively smaller magnitudes for developmental effects and relatively larger magnitudes in intervention contexts [55]. Cohen’s |*d*s| of the pandemic effects range between 0.02 for powerLOW and 0.15 for coordination. The second row of Table 5 shows the effects of the pandemic in the original test metric, before z-score computation. These estimates were computed in separate LMMs for each physical fitness component with score (instead of z-score) as the dependent variable. The LMMs took into account effects of sex, age, and five cohort indicator variables and included the random factor school, with school-related VCs for sex, age, the five cohort indicator variables (for the full results of these LMMs, see the script *keyage_lmm_16_22.qmd* in the OSF repository).

In sports science, the smallest meaningful change (SMC) indicates the minimum size an effect must have to be interpreted as practically relevant [56]. When SMC is computed with 0.2*SD, all Covid pandemic effects are smaller than the corresponding SMCs. According to this metric, the observed Covid pandemic effects appear not to be practically relevant.

In educational sciences, the relevance of an effect is indicated by how many months children are advanced in or behind their expected development. We computed the months of developmental costs/gains related to the Covid pandemic by $\frac{{\text{Covid}\, \text{effect}}}{{\text{Age}\, \text{effect}}}*12$ relative to a longitudinal 1-year development. The 1-year physical fitness development was available from a sample of 1013 keyage children from 31 schools who were tested in third grade and retested one year later in fourth grade as part of the longitudinal arm of the EMOTIKON study [34]. The longitudinal age effect was computed in LMMs that took into account the effect of sex and the development-related VCs and CPs for the random factors child and school (for the full results of these LMMs, see *keyage_devel_costs.Rmd* in the OSF repository). When comparing performance in cohorts 2020 until 2022 to the cohorts 2016 until 2019, children in the 2020–2022 cohorts exhibited developmental delays of approximately 5 months in cardiorespiratory endurance and 3 months in coordination. Children were 1 and 2 months behind in their speed and powerUP development, respectively. They were approximately 1 month in advance in their development of powerLOW, and close to 7 months in advance in their development of static balance.

### Linear Mixed Model for Older-than-Keyage Children

In LMMs for OTK children, the dependent variable was *delta z-scores* (i.e., observed z-scores–predicted z-scores). *Delta z-scores* indicate that the observed test performance fell short of the predicted performance (i.e., negative *delta z-score*) or was higher than predicted (i.e., positive *delta z-score*). A significant negative Covid pandemic effect (two-level Covid factor comparing *delta z-scores* in 2016–2019 with *delta z-scores* in 2020–2022) indicates that OTK children were more affected by the Covid pandemic than keyage children. Table 6 shows fixed effect estimates, standard errors, and z-values of the LMM for OTK children. *Delta z-scores* were larger for the star-run test than for the 6-min run test (*b* = 0.268, z = 2.81) and larger for the ball-push test than for the one-legged stance test (*b* = − 0.356, z = − 3.24).

**Table 6:** Fixed effect estimates, standard errors and z-values of the LMM for OTK children

Source of variance	Fixed-effect estimate	Standard error	z-value
Grand mean (intercept)	0.433	0.054	**7.98**
Physical fitness component			
ECS versus LUB	0.079	0.058	1.37
EC versus S	− 0.111	0.076	− 1.46
E versus C	0.268	0.095	**2.81**
L versus U	0.133	0.097	1.38
U versus B	− 0.356	0.110	− **3.24**
Covid comparison (2016–2019 versus 2020–2022) nested within physical fitness components			
Endurance: Covid	− 0.038	0.014	− **2.82**
Coordination: Covid	− 0.024	0.014	− 1.75
Speed: Covid	− 0.008	0.013	− 0.57
PowerLOW: Covid	− 0.018	0.014	− 1.28
PowerUP: Covid	− 0.027	0.013	− **2.05**
Balance: Covid	− 0.039	0.013	− **3.02**
Age (linear) nested within physical fitness components			
Endurance: a1	− 0.737	0.187	− **3.94**
Coordination: a1	− 1.436	0.187	− **7.70**
Speed: a1	− 0.793	0.185	− **4.28**
PowerLOW: a1	− 1.254	0.193	− **6.51**
PowerUP: a1	− 1.594	0.184	− **8.67**
Balance: a1	− 0.695	0.175	− **3.98**
Age (quadratic) nested within physical fitness components			
Endurance: a2	0.206	0.097	**2.12**
Coordination: a2	0.507	0.097	**5.25**
Speed: a2	0.229	0.096	**2.39**
PowerLOW: a2	0.433	0.100	**4.34**
PowerUP: a2	0.643	0.095	**6.74**
Balance: a2	0.228	0.091	**2.51**
Sex nested within physical fitness components			
Endurance: Sex	− 0.038	0.013	− **3.06**
Coordination: Sex	0.051	0.013	**4.08**
Speed: Sex	0.018	0.012	1.46
PowerLOW: Sex	0.070	0.013	**5.45**
PowerUP: Sex	0.051	0.012	**4.14**
Balance: Sex	0.060	0.012	**5.12**

Physical fitness contrasts: ECS versus LUB = endurance, coordination, and speed versus powerLOW, powerUP, and balance. EC versus S = endurance and coordination versus speed. E versus C = endurance versus coordination. L versus U = powerLOW versus powerUP. U versus B = powerUP versus balance. Covid = Comparison of cohorts 2016–2019 with cohorts 2020–2022. Endurance = cardiorespiratory endurance (i.e., 6-min run test), coordination = star-run test, speed = 20-m linear sprint test, powerLOW = lower limbs muscle power (i.e., standing long jump test), powerUP = upper limbs muscle power (i.e., ball-push test), Balance = static balance (i.e., one-legged-stance test with eyes closed). Bold = |z| > 2.0, linear mixed model random factors: schools (514) and children (27,383), observations = 157,751. For estimates of variance components and correlation parameters, see Table 7

**Fig. 2:**
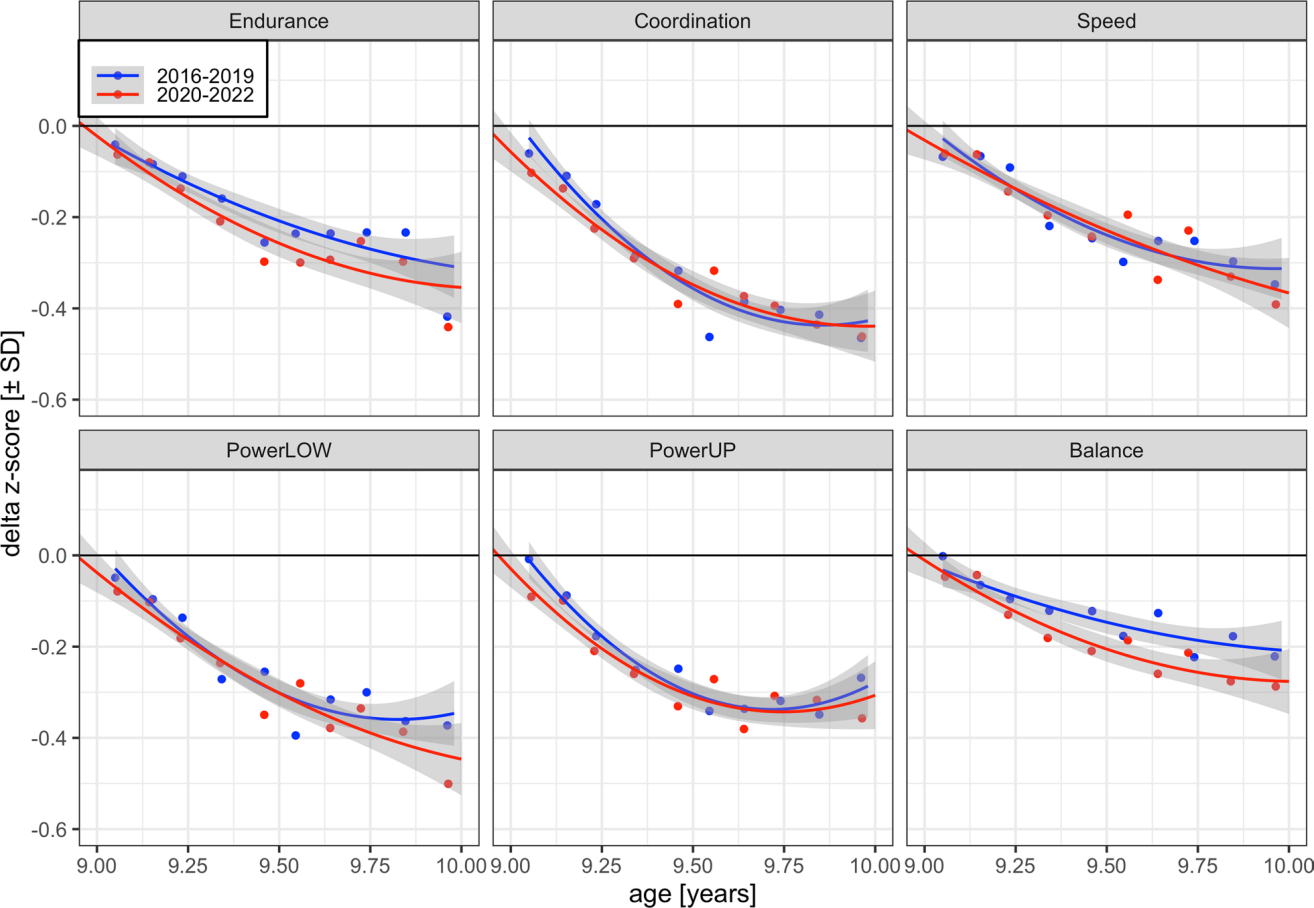
*Delta z-scores* (± SE) of OTK third-graders between 9 and 10 years before (cohorts 2016–2019, blue) and during or after (cohorts 2020–2022, red) the Covid pandemic. Points are binned *delta* child means. Endurance = cardiorespiratory endurance (i.e., 6-min-run test), Coordination = star-run test, Speed = 20-m linear sprint test, PowerLOW = lower limbs muscle power (i.e., standing long jump test), PowerUP = upper limbs muscle power (i.e., ball-push test), balance = static balance (i.e., one-legged-stance test with eyes closed). For coordination and speed, scores were converted from seconds to meters/seconds (i.e., pace scores; star-run test = 50.912 [m]/time [s]; 20-m linear sprint test = 20 [m]/time [s]). For static balance, scores were log-transformed

Interestingly, we found evidence for OTK-specific negative Covid pandemic effects. The Covid-19 pandemic further increased the OTK children’s deviations between observed and predicted performance in the 6-min run test (*b* = − 0.038, z = − 2.82), ball-push test (*b* = − 0.027, z = − 2.05), and one-legged stance test (*b* = − 0.039, z = − 3.02). Figure 2 illustrates these effects. OTK children during and after the pandemic (cohorts 2020 and 2022, shown in red) exhibited slightly more negative *delta z-scores* than OTK children before the pandemic (cohorts 2016–2019, shown in blue) in tests of cardiorespiratory endurance, powerUP and balance.

As reported by Fühner et al. [33], there was a negative linear age effect on the *delta z-scores* of all physical fitness tests (*b*s between − 1.594 for the ball-push test and − 0.695 for the one-legged stance test, *z* between − 8.67 for the ball-push test and − 3.94 for the 6-min run test). This indicates that physical fitness deficits of OTK children increased with increasing age. The linear negative age effect on *delta-z-score* was followed by a plateau for all six physical fitness tests (*b*s between 0.206 for the 6-min run test and 0.643 for the ball-push test, *z* between 2.12 for 6-min run test and 6.74 for the ball-push test).

OTK girls, compared to OTK boys, showed larger deviations between observed and predicted performance (their *delta z-score* was more negative) in the star-run test (*b* = 0.051, z = 4.08), standing long jump test (*b* = 0.070, z = 5.45), ball-push test (*b* = 0.051, z = 4.14) and the one-legged stance test (*b* = 0.060, z = 5.12), while OTK boys were more impaired than OTK girls in the 6-min run test performance (*b* = − 0.038, z = − 3.06).

The VCs and CPs for the random factors child and school are shown in Table 7. Physical fitness component-related differences were larger between children (VCs between 0.528 and 0.736) than between schools (VCs between 0.002 and 0.007). The *delta z-scores* of the physical fitness tests correlated positively with each other after statistical adjustment of all the other effects, indicating that children with larger deviations between predicted and observed performance in one physical fitness test likely also exhibited larger deviations between predicted and observed performance in the other tests. The *delta z-scores* of the four tests assessing cardiorespiratory endurance, coordination, speed, and powerLOW correlated highly with each other (CPs between 0.57 and 0.82), whereas the correlations of the *delta z-scores* of the tests assessing powerUP and balance with the other four tests were smaller (CPs between 0.23 and 0.53). Differences between schools in the linear age and Covid effect were small (VCs 0.004 and 0.011, respectively) but significant.

**Table 7:** Child- and school-related variance components and correlation parameters of the LMM for OTK children

	VC	CP					
		Endurance	Coord	Speed	PowerLOW	PowerUP	Balance
Child							
Endurance	0.653	1.00					
Coord	0.649	0.57	1.00				
Speed	0.649	0.66	0.68	1.00			
PowerLOW	0.736	0.63	0.69	0.82	1.00		
PowerUP	0.636	0.23	0.48	0.45	0.53	1.00	
Balance	0.528	0.26	0.30	0.31	0.34	0.12	1.00
School							
Endurance	0.006						
Coord	0.007	–					
Speed	0.002	–	–				
PowerLOW	0.003	–	–	–			
PowerUP	0.006	–	–	–	–		
Balance	0.007	–	–	–	–	–	
Covid	0.011	–	–	–	–	–	–
Age (linear)	0.004	–	–	–	–	–	–

Endurance = cardiorespiratory endurance (i.e., 6-min run test), Coord = coordination (i.e., star run test), Speed = 20-m linear sprint test, PowerLOW = lower limbs muscle power (i.e., standing long jump test), PowerUP = upper limbs muscle power (i.e., ball-push test), balance = static balance (i.e., one-legged-stance test with eyes closed). VC = variance component, CP = correlation parameter. VC for Residual = 0.341

## Discussion

Taking advantage of annual fitness tests since 2016, we tested potential Covid-19 pandemic effects on the physical fitness of German third-graders in a state-wide assessment in the Federal State of Brandenburg, Germany. We used linear mixed models to compare physical fitness in the pandemic cohorts 2020–2022 with physical fitness in the pre-pandemic cohorts 2016–2019.

When comparing the pandemic cohorts 2020 until 2022 with pre-pandemic cohorts 2016 until 2019, we found changes in all six assessed physical fitness components. The Covid pandemic was associated with decreased performance in three running tests (i.e., cardiorespiratory endurance, coordination, speed), as well as in powerUP. PowerLOW and static balance were better after the start of the Covid pandemic compared to the pre-pandemic cohorts. Although pandemic-related changes were small (Cohen’s |*d*s| between 0.02 for powerLOW and 0.15 for coordination) and did not exceed the threshold for the SMC, they were associated with developmental costs, or, in the case of powerLOW and static balance, developmental gains, of several months. Children in cohorts 2020 to 2022 were estimated to exhibit developmental delays of approximately 5 months in cardiorespiratory endurance, 3 months in coordination, and 1 and 2 months in speed and powerUP, respectively. They were approximately 1 month in advance in their powerLOW development and 7 months in advance in their development of static balance.

We also tested whether children’s physical fitness changed from the first to second pandemic year, and whether we would observe potential ‘rebound’ effects after the pandemic. Interestingly, children’s performance further declined after 2020 in coordination and powerUP. For powerLOW, children initially showed a positive pandemic effect, but performance also declined from 2020 to 2021. With the exception of powerUP, we found no evidence of physical fitness improvements after the pandemic. Why do we not see much of a difference between the pandemic cohorts? One explanation could be that in the year 2020, in which the strictest social distancing measures including repeated school closures were implemented [57, 58], the 2020, 2021, and 2022 cohorts were in grades 3, 2, and 1, respectively. The 2021 and 2022 cohorts may simply have been unable to compensate this loss of structured physical exercise in their first or second school year. Children who will enter third grade in the school year 2023/24 are the first ones not to have experienced the first pandemic year as school children, as they were still in kindergarten in 2020. Data from future cohorts are needed to test whether there will be a ‘catch-up’ of physical fitness.

When comparing physical fitness across different cohorts, secular trends need to be considered. Could pre-pandemic secular trends between 2016 and 2019 account for the observed physical fitness changes during the pandemic years? As shown in Fig. 1, the three run tasks exhibit a qualitative negative discontinuity between assessments in the falls of 2019 and 2020, but their profiles for pre-pandemic and pandemic years are very different from each other.

Speed performance was characterized by a pre-pandemic linear increase, which had already been reported in a previous study including the same data from pre-pandemic cohorts [32]. There was a discontinuous decrement between the assessments in falls of 2019 and 2020. We can extrapolate the pandemic decline back to the first school day after the summer holidays on August 10, 2020 using a regression discontinuity design (RDD) [59, 60]. In a post-hoc RDD-LMM (see RDD analysis in OSF repository) the Covid effect was significant not only for the difference between all pre-pandemic and pandemic assessments, but also at that specific day, and sprint performance decreased during the pandemic years.

Coordination performance declined to a plateau during pre-pandemic years, followed by a large discontinuous decrement between assessments in falls of 2019 and 2020, and low levels of performance during pandemic years. In the RDD-LMM, the decrement was also significant at the first school day after the summer break 2020.

For performance in cardiorespiratory endurance, no secular trend was detected during pre-pandemic years. Again, there is a discontinuous decrement between assessments in falls of 2019 and 2020. As was the case for the other two run-tests, this effect was also significant at the first school day after the summer break 2020 tested in the RDD-LMM. However, using data from 2011 until 2022, cardiorespiratory endurance declined linearly in the pre-pandemic cohorts (see Additional file 1: Fig. S1). Thus, the negative pandemic effect reported above possibly overestimates Covid-related changes in cardiorespiratory endurance.

The performance profiles were also qualitatively different for the three non-running tasks. For powerLOW and static balance, performance was better and for powerUP worse during pandemic compared to pre-pandemic years. However, as shown in Fig. 1, for powerUP and balance there is not much evidence for a discontinuity between fall assessments of 2019 and 2020. In the RDD-LMM, neither of the Covid effects was significant at the cut-off day and this does not depend on the choice of the cut-off day between assessments. The powerLOW profile suggests a temporary elevation of performance in 2020. However, as performance was already increasing prior to the pandemic, the Covid effect on powerLOW was not significant when tested on August 10, 2020, in the post-hoc RDD-LMM. Changes in powerLOW, powerUP, and static balance are thus likely due to pandemic-independent secular cohort trends.

One explanation for the negative pandemic effects on the three run tests (i.e., 6-min run, star-run, 20-m sprint test) and possibly the increases in powerLOW (i.e., standing long jump test) and balance (i.e., one-legged stance test) is that muscle-strengthening tasks and tasks enhancing static balance can be practiced in small, confined spaces. In contrast, running tasks require larger spaces that were hardly accessible during repeated periods of homeschooling and social distancing measures. The Covid-19 pandemic effect in standardized z-score units was largest for coordination. Coordination was assessed using the star-run test, in which children had to memorize a star-like pattern associated with different movement forms to be carried out in a specific order. The cognitive load of the star-run test is higher than for the other physical fitness tests. A decline in performance in this test might not only indicate a Covid pandemic “cost” in physical coordination skills, but also in working memory. In agreement with this hypothesis, other studies reported decreases in children’s executive function [19] and academic learning losses in mathematics, reading and spelling [61–63] during the pandemic.

Our results are also in line with other studies reporting pandemic-related declines of children’s and adolescents’ cardiorespiratory endurance [19–21, 23–26, 64] and speed [22, 24, 28, 64, 65]. For powerLOW, Chambonnière et al. [19] report a pandemic-related performance decline of third- and fourth-graders in the standing long jump. In contrast, our study as well as other German studies [22, 25], found a pandemic-related increase in the standing long jump test performance. One factor possibly associated with the differences between results is the different assessment times. Whereas we and two other studies [22, 25] tested the first pandemic cohort in fall and early winter of 2020, Chambonnière et al. [19] tested the pandemic cohort in January of 2021 after a lockdown in France, where children may have had even fewer outdoor opportunities to compensate for movement restrictions. While the present study, along with studies from France [19] and Austria [26], found an initial pandemic-related decline in upper limbs muscle power, another Austrian study reported higher upper limbs muscle power in 2022 compared to pre-pandemic cohorts [21]. In the present study, the initial decline of powerUP after the start of the pandemic was followed by an increase of performance from 2021 to 2022.

An important question that has been raised in other contexts, such as psychological health [66, 67], physical activity levels [68, 69], and academic learning losses [61–63, 70] is whether the Covid pandemic decreased or increased social inequalities. Interestingly, we found evidence for the both hypotheses. On the one hand, the pandemic exacerbated the physical fitness delays of OTK children, who had already exhibited physical fitness deficits before the pandemic [33]. In the 2020- to-2022 cohorts, OTK children fell further behind in cardiorespiratory endurance, powerUP, and balance. While we do not know the socioeconomic status or the reason for the delayed school enrollment of OTK children in our sample, research indicates that delayed school enrollment, at least for a subgroup of OTK children, may be associated with socioeconomic disadvantage [71, 72]. Certain groups of disadvantaged children might have had fewer resources available to compensate for the loss of structured physical activity and were more negatively affected by the pandemic. Similar to our results, Wessely et al. [25] reported that pandemic-related losses were higher among children with a high social burden, who already exhibited a lower pre-pandemic physical fitness compared to children with a low social burden. On the other hand, the RDD analyses reported in the OSF repository showed a negative correlation between the schools’ random intercept and the Covid pandemic effect: “Fitter” schools (larger conditional modes for the Grand Mean) thus exhibited larger negative Covid pandemic effects. A similar pattern of results, but regarding physical activity levels, was reported in Croatia, where adolescents living in urban areas exhibited higher pre-pandemic physical activity levels and showed a larger decline in their physical activity than adolescents living in rural areas [73]. According to the authors, adolescents in urban areas had more access to organized sports than rural adolescents before the pandemic, and the pandemic-related restrictions thus had a larger effect on the physical activity levels of urban, compared to rural adolescents. The results reported above are not mutually exclusive. It is possible that at the individual level, certain groups of disadvantaged children might have had fewer resources available to compensate for the loss of structured physical activity and were more negatively affected by the pandemic. At the same time, at the school level, fitter schools with highly active children and possibly located in more affluent regions with a larger number of options to join organized sports activities had more to lose by the pandemic-related restrictions than schools of children with lower pre-pandemic physical fitness levels; they thus showed a higher pandemic-related drop in physical fitness.

Aside from Covid pandemic effects, our analyses replicated age and sex effects reported previously [32] and added information on a sixth physical fitness component, that is static balance. The cross-sectional age effects of keyage children were linear for boys and girls for each of the six physical fitness components. Boys outperformed girls in all physical fitness tests with the exception of the one-legged stance test, where girls outperformed boys. The better static balance of girls is in line with results from previous research reporting gender differences in the development of postural control [74–77]. Better static balance of girls in the ninth year of life might be related to a faster maturation of the vestibular system [78, 79] and a faster development of sensory integration ability [79, 80] in girls. Previous research reported a higher reliance on visual information for postural control in preadolescent boys compared to girls [74], and our results may thus indicate a better sensory reweighting ability in girls compared to boys in the ninth year of life.

As reported by Fühner et al. [33], OTK children’s physical fitness fell short of the physical fitness that could be expected for their age, and the difference between predicted and observed physical fitness increased with increasing age. The previous report found sex differences in the deviations of observed from expected performance only in cardiorespiratory endurance and powerLOW. In the present study, we replicated the findings that OTK boys showed larger deviations from their expected performance than OTK girls in cardiorespiratory endurance, and OTK girls showed greater deficits than OTK boys in powerLOW. However, we found additional sex differences in coordination, powerUP and balance, where OTK girls were more impaired than OTK boys.

We also replicated high correlations between performances in 6-min run, star-run, 20-m sprint, and the standing long jump tests reported by Fühner et al. [32]. The correlations of the ball-push test and the newly added one-legged stance test with the other four tests were lower. The first four physical fitness components thus clearly represent a latent construct of *physical fitness*. The low correlation between the one-legged stance with the other tests may be explained by the fact that performance in a static balance test is not energetically-driven, but, as mentioned above, reflects differences in sensory integration and reweighting abilities [79, 81]. This result is in line with previous research reporting no significant association of balance and muscular strength in children [82, 83]. The ball-push test, on the other hand, is the only one out of the six tests in which overweight children outperform normal weight children [84] and might thus also be an indicator of physical ‘unfitness’. These results should be considered when assembling an economical test battery to assess children’s physical fitness, especially since assessments conducted in schools are almost always associated with time constraints. Physical fitness tests assessing cardiorespiratory endurance, coordination, speed, and powerLOW likely have the highest relevance and may be prioritized, whereas the ball-push test and the one-legged stance test with eyes closed might be considered with a lower priority.

Our study is not without limitations. First, we do not have objective anthropometric information. In 2021 and 2022, a subset of parents voluntarily provided information about body mass and body height. However, these data do not allow us to control for secular trends and to assess associations with Covid pandemic effects. Several studies report increases in children’s and adolescents’ BMI during the pandemic [20, 21, 25, 26, 85]. Increases in body mass negatively affect performance in weight-bearing tests [84] and may be associated with the negative pandemic effects on the three running tests in the present study. However, other studies report no evidence for changes in BMI [19, 24, 86] or subgroup-specific changes depending on gender [23, 26], socioeconomic status [25, 85], or comorbidities [87].

A second limitation relates to the dissociation of long-term cohort-related and short-term Covid pandemic-related effects. Our results are contingent on the set of contrasts chosen for the statistical model. In the OSF repository, we report results from an LMM implementing a regression discontinuity design (RDD) [59, 60]. Specifically, we tested (a) whether the Covid pandemic effects were significant at the first day of the school year in August 2020 (rather than the mean difference between pre-pandemic and pandemic cohorts) and (b) whether secular cohort effects were different before and after this day. These results were largely in agreement with interpretations based on the primary LMM. Other cut-off days may yield different results; data and scripts are available for re-analyses in the OSF repository. By definition, quasi-experimental studies do not afford strong causal inferences and their limitations are well documented [59, 60].

A third limitation is that we do not have individual information on the socioeconomic background, living environment, or physical activity levels of the children in our sample. Previous studies have shown that access to an outdoor area like an own garden [19, 69], household income [68], and living in a house versus an apartment [68] were positively associated with physical activity levels during the pandemic. It is also likely that some families of children in our sample were able to compensate for movement restrictions better than others and that some schools were able to implement online or outdoor exercise programs during lockdowns, while other schools were not, resulting in differential Covid pandemic effects on children’s physical fitness. Future analyses integrating community- or school-based social indices could yield more detailed information about factors associated with pandemic-related changes in children’s physical fitness and its post-pandemic development.

## Conclusions

We tested Covid-19 pandemic effects on the physical fitness of children using a large, representative sample of German third-graders. Children exhibited lower cardiorespiratory endurance, coordination, speed and powerUP in the Covid pandemic cohorts (2020–2022) compared to the pre-pandemic cohorts (2016–2019). Children’s powerLOW and static balance were higher in the pandemic cohorts compared to the pre-pandemic cohorts. Pre-pandemic secular trends may account for some of the physical fitness changes observed during the pandemic, especially in powerLOW, powerUP and balance. Learning losses of several months should be met with concern, especially in light of the associations between physical fitness and physical health [5, 6], psychological well-being [8], and cognitive function [9, 10]. School- or community-based exercise programs to improve children’s physical fitness may particularly target those fitness components that were negatively affected by the pandemic. Programs may aim to enhance the quality and quantity of school sports [88, 89], encourage active commutes to school [90], develop organized sports structures, or create outdoor spaces to exercise. An important goal is to increase access to sports opportunities for socioeconomically deprived children. Future annual EMOTIKON assessments will monitor children’s physical fitness and examine whether children will catch up or whether the negative Covid-19 pandemic effects further accumulate across cohorts.

### Supplementary Information

**Additional file 1.** Supplementary Tables and Figure.

## Data Availability

Data as well as R and Julia scripts are available in the Open Science Framework (OSF) repository: https://osf.io/w975d/ [39].

## Abbreviations

ECS versus LUB: Cardiorespiratory endurance, coordination and speed versus powerLOW, powerUP, and static balance
EC versus S: Cardiorespiratory endurance and coordination versus speed
E versus C: Cardiorespiratory endurance versus coordination
L versus U: PowerLOW versus powerUP
U versus B: PowerUP versus static balance
CP: Correlation parameter
LMM: Linear mixed model
OTK: Older-than-keyage
PowerLOW: Lower limbs muscle power
PowerUP: Upper limbs muscle power
SMC: Smallest meaningful change
VC: Variance component
