# Supplementary material for: Covid Pandemic Effects on the Physical Fitness of Primary School Children: Results of the German EMOTIKON Project
Source: Sports Med Open. 2023 Aug 14;9:77. doi: 10.1186/s40798-023-00624-1 (PMC10425322; doi:10.1186/s40798-023-00624-1)
Supplement: Supplementary file 1 — Additional file 1. Supplementary Tables and Figure. [file 40798_2023_624_MOESM1_ESM.pdf]

Covid Pandemic Effects on the Physical Fitness of Primary School Children: Results of the German EMOTIKON Project

Paula Teich, Thea Fühner, Florian Bähr, Christian Puta, Urs Granacher, Reinhold Kliegl

Supplementary Material

Sample description for keyage and for older-than-keyage children

Table S1. Sample description for keyage children

PF Component	*Cohorts*	*Sex*	*N Children*	*N Schools*	*Age in years Mean (SD)*	*Test score Mean (SD)*
Endurance	pre (2016-19)	Boys	25,492	498	8.63 (0.28)	1031.8 m (163.9)
	pre (2016-19)	Girls	26,642	495	8.60 (0.28)	962.3 m (139.4)
	peri (2020+21)	Boys	13,450	485	8.56 (0.27)	1016.2 m (164.7)
	peri (2020+21)	Girls	14,127	480	8.53 (0.28)	952.0 m (141.9)
	post (2022)	Boys	6,915	466	8.60 (0.27)	1016.2 m (166.7)
	post (2022)	Girls	7,414	461	8.57 (0.28)	944.9 m (144.2)
Coordination	pre (2016-19)	Boys	25,383	498	8.63 (0.28)	2.08 m/s (0.30)
	pre (2016-19)	Girls	26,558	495	8.60 (0.28)	2.01 m/s (0.27)
	peri (2020+21)	Boys	13,472	487	8.56 (0.27)	2.04 m/s (0.31)
	peri (2020+21)	Girls	14,209	481	8.53 (0.28)	1.97 m/s (0.29)
	post (2022)	Boys	6,883	465	8.60 (0.27)	2.04 m/s (0.32)
	post (2022)	Girls	7,449	461	8.57 (0.28)	1.97 m/s (0.29)
Speed	pre (2016-19)	Boys	25,571	497	8.63 (0.28)	4.62 m/s (0.43)
	pre (2016-19)	Girls	26,689	495	8.60 (0.28)	4.49 m/s (0.40)
	peri (2020+21)	Boys	13,776	486	8.56 (0.27)	4.59 m/s (0.44)
	peri (2020+21)	Girls	14,458	479	8.53 (0.28)	4.47 m/s (0.42)
	post (2022)	Boys	7,016	466	8.60 (0.27)	4.59 m/s (0.44)
	post (2022)	Girls	7,546	463	8.57 (0.28)	4.46 m/s (0.42)
PowerLOW	pre (2016-19)	Boys	25,865	499	8.63 (0.28)	129.2 cm (19.8)
	pre (2016-19)	Girls	27,013	494	8.60 (0.28)	121.7 cm (18.6)
	peri (2020+21)	Boys	13,879	488	8.56 (0.27)	129.7 cm (20.2)
	peri (2020+21)	Girls	14,535	481	8.53 (0.28)	121.8 cm (18.6)
	post (2022)	Boys	6,981	466	8.60 (0.27)	129.6 cm (19.9)
	post (2022)	Girls	7,530	462	8.57 (0.28)	121.6 cm (18.9)
PowerUP	pre (2016-19)	Boys	26,079	500	8.63 (0.28)	4.0 m (0.7)
	pre (2016-19)	Girls	27,299	497	8.60 (0.28)	3.5 m (0.7)

PF Component	*Cohorts*	*Sex*	*N Children*	*N Schools*	*Age in years Mean (SD)*	*Test score Mean (SD)*
PowerUP	peri (2020+21)	Boys	13,901	488	8.56 (0.27)	3.9 m (0.7)
	peri (2020+21)	Girls	14,571	481	8.53 (0.28)	3.4 m (0.6)
	post (2022)	Boys	7,060	466	8.60 (0.27)	4.0 m (0.7)
	post (2022)	Girls	7,603	463	8.57 (0.28)	3.5 m (0.6)
Balance	pre (2016-19)	Boys	25,687	499	8.63 (0.28)	17.7 s (16.2)
	pre (2016-19)	Girls	26,982	497	8.60 (0.28)	21.6 s (18.1)
	peri (2020+21)	Boys	13,747	488	8.56 (0.27)	19.3 s (17.3)
	peri (2020+21)	Girls	14,468	481	8.53 (0.28)	23.4 s (18.9)
	post (2022)	Boys	6,968	469	8.60 (0.27)	19.5 s (17.6)
	post (2022)	Girls	7,568	465	8.57 (0.28)	23.5 s (19.1)

PF Component = Physical fitness component. Endurance = cardiorespiratory endurance (i.e., 6-min run test), coordination = star-run test, speed = 20-m linear sprint test, powerLOW = lower limbs muscle power (i.e., standing long jump test), powerUP = upper limbs muscle power (i.e., ball-push test), Balance = one-legged stance test with eyes closed. Cohorts: pre 2016-19 = pre-pandemic cohorts, peri 2020+21 = peri-pandemic cohorts, post (2022) = post-pandemic cohort.

Table S2. Sample description for older-than-keyage children (OTK)

PF Component	*Cohorts*	*Sex*	*N Children*	*N Schools*	*Age in years Mean (SD)*	*Test scores Mean (SD)*
**Endurance**	pre (2016-19)	Boys	7,968	496	9.38 (0.24)	1006.6 m (175.5)
	pre (2016-19)	Girls	5,643	486	9.38 (0.25)	941.1 m (148.0)
	peri (2020+21)	Boys	4,637	483	9.32 (0.26)	988.0 m (178.4)
	peri (2020+21)	Girls	3,347	475	9.33 (0.27)	928.0 m (152.2)
	post (2022)	Boys	2,543	452	9.36 (0.26)	989.7 m (178.6)
	post (2022)	Girls	1,817	432	9.34 (0.26)	928.8 m (154.6)
Coordination	pre (2016-19)	Boys	7,924	496	9.38 (0.25)	2.07 m/s (0.32)
	pre (2016-19)	Girls	5,616	485	9.38 (0.25)	1.99 m/s (0.30)
	peri (2020+21)	Boys	4,666	486	9.32 (0.26)	2.02 m/s (0.33)
	peri (2020+21)	Girls	3,398	477	9.33 (0.27)	1.94 m/s (0.32)
	post (2022)	Boys	2,549	449	9.36 (0.26)	2.03 m/s (0.33)
	post (2022)	Girls	1,838	429	9.35 (0.26)	1.94 m/s (0.31)
Speed	pre (2016-19)	Boys	7,981	495	9.38 (0.25)	4.60 m/s (0.46)
	pre (2016-19)	Girls	5,654	485	9.38 (0.25)	4.46 m/s (0.42)
	peri (2020+21)	Boys	4,770	483	9.33 (0.26)	4.58 m/s (0.47)
	peri (2020+21)	Girls	3,434	475	9.34 (0.27)	4.45 m/s (0.44)
	post (2022)	Boys	2,595	451	9.36 (0.26)	4.57 m/s (0.47)
	post (2022)	Girls	1,856	433	9.34 (0.26)	4.44 m/s (0.45)
PowerLOW	pre (2016-19)	Boys	8,078	496	9.38 (0.25)	127.6 cm (21.6)
	pre (2016-19)	Girls	5,689	486	9.38 (0.25)	119.3 cm (19.8)
	peri (2020+21)	Boys	4,817	486	9.32 (0.26)	128.5 cm (21.9)
	peri (2020+21)	Girls	3,443	476	9.34 (0.27)	119.2 cm (20.0)
	post (2022)	Boys	2,589	450	9.36 (0.26)	128.5 cm (21.8)
	post (2022)	Girls	1,866	433	9.35 (0.26)	118.7 cm (20.6)
PowerUP	pre (2016-19)	Boys	8,188	496	9.38 (0.25)	4.2 m (0.78)
	pre (2016-19)	Girls	5,742	486	9.38 (0.25)	3.6 m (0.71)
	peri (2020+21)	Boys	4,799	487	9.32 (0.26)	4.1 m (0.78)
	peri (2020+21)	Girls	3,464	476	9.34 (0.27)	3.6 m (0.69)

PF Component	*Cohorts*	*Sex*	*N Children*	*N Schools*	*Age in years Mean (SD)*	*Test scores Mean (SD)*
PowerUP	post (2022)	Boys	2,611	451	9.36 (0.26)	4.1 m (0.77)
	post (2022)	Girls	1,879	434	9.35 (0.26)	3.6 m (0.70)
Balance	pre (2016-19)	Boys	8,019	495	9.38 (0.25)	17.7 s (16.3)
	pre (2016-19)	Girls	5,648	484	9.38 (0.25)	21.2 s (18.0)
	peri (2020+21)	Boys	4,768	488	9.32 (0.27)	19.3 s (17.6)
	peri (2020+21)	Girls	3,458	476	9.34 (0.27)	22.1 s (18.7)
	post (2022)	Boys	2,585	453	9.36 (0.26)	19.3 s (17.4)
	post (2022)	Girls	1,872	435	9.35 (0.26)	22.8 s (18.8)

PF Component = Physical fitness component. Endurance = cardiorespiratory endurance (i.e., 6-min run test), coordination = star-run test, speed = 20-m linear sprint test, powerLOW = lower limbs muscle power (i.e., standing long jump test), powerUP = upper limbs muscle power (i.e., ball-push test), Balance = one-legged stance test with eyes closed. Cohorts: pre 2016-19 = pre-pandemic cohorts, peri 2020+21 = peri-pandemic cohorts, post (2022) = post-pandemic cohort.

Reparameterized linear mixed model for keyage children in cohorts 2016 until 2022 with physical fitness component levels instead of contrasts

The reparameterized version of the LMM for keyage children includes physical fitness component levels instead of physical fitness component contrasts. Table S3 shows the fixed effect estimates, standard errors and z-values of this LMM. Table S4 shows the child- and school-related variance components and correlation parameters.

Table S3. Fixed effect estimates, standard errors and z-values of reparameterized LMM for keyage children

Source of variance	Fixed-effect estimate	Standard error	z-value
Physical fitness component			
Endurance	-0.062	0.017	**-3.76**
Coordination	-0.051	0.018	**-2.81**
Speed	-0.062	0.016	**-3.97**
PowerLOW	-0.050	0.013	**-3.87**
PowerUP	-0.043	0.012	**-3.69**
Balance	0.002	0.016	0.12
**Cohort contrasts**			
Endurance *(6-min run test)*			
Pre1 (linear)	-0.029	0.061	-0.48
Pre2 (quadratic)	0.014	0.025	0.56
Covid contrast 1	-0.077	0.011	**-7.32**
Covid contrast 2	-0.022	0.016	-1.35
Covid contrast 3	-0.011	0.016	-0.65
Coordination *(star-run test)*			
Pre1 (linear)	-0.060	0.061	-0.98
Pre2 (quadratic)	0.093	0.025	**3.71**
Covid contrast 1	-0.146	0.011	**-13.79**
Covid contrast 2	-0.037	0.016	**-2.29**
Covid contrast 3	0.025	0.016	1.55
Speed *(20-m sprint test)*			
Pre1 (linear)	0.230	0.061	**3.76**
Pre2 (quadratic)	0.032	0.025	1.26
Covid contrast 1	-0.029	0.011	**-2.78**
Covid contrast 2	-0.030	0.016	-1.87
Covid contrast 3	0.013	0.016	0.82
PowerLOW *(standing long jump test)*			
Pre1 (linear)	-0.085	0.062	-1.38
Pre2 (quadratic)	0.070	0.025	**2.78**
Covid contrast 1	0.022	0.011	**2.06**
Covid contrast 2	-0.039	0.016	**-2.39**
Covid contrast 3	0.006	0.016	0.38

Source of variance	Fixed-effect estimate	Standard error	z-value
**PowerUP** *(ball-push test)*			
Pre1 (linear)	-0.241	0.060	**-4.00**
Pre2 (quadratic)	0.064	0.025	**2.61**
Covid contrast 1	-0.083	0.010	**-7.96**
Covid contrast 2	-0.065	0.016	**-4.07**
Covid contrast 3	0.044	0.016	**2.78**
**Balance** *(one-legged-stance test)*			
Pre1 (linear)	0.031	0.061	0.51
Pre2 (quadratic)	0.068	0.025	**2.71**
Covid contrast 1	0.080	0.011	**7.58**
Covid contrast 2	0.024	0.016	1.48
Covid contrast 3	0.003	0.016	0.18
Age (linear) nested within physical fitness component			
Endurance: a1	0.072	0.011	**6.58**
Coordination: a1	0.274	0.011	**25.04**
Speed: a1	0.202	0.011	**18.38**
PowerLOW: a1	0.209	0.011	**18.62**
PowerUP: a1	0.516	0.011	**49.16**
Balance: a1	0.132	0.011	**12.13**
Sex nested within physical fitness component			
Endurance: Sex	0.435	0.006	**69.31**
Coordination: Sex	0.225	0.006	**35.65**
Speed: Sex	0.296	0.006	**46.83**
PowerLOW: Sex	0.380	0.007	**58.77**
PowerUP: Sex	0.663	0.006	**109.26**
Balance: Sex	-0.244	0.006	**-39.00**

Endurance = cardiorespiratory endurance (i.e., 6-min run test), coordination = star-run test, speed = 20-m linear sprint test, powerLOW = lower limbs muscle power (i.e., standing long jump test), powerUP = upper limbs muscle power (i.e., ball-push test), Balance = one-legged stance test with eyes closed. Cohort contrasts: Pre1 (linear) = Linear pre-pandemic secular trend. Pre2 (quadratic) = Quadratic pre-pandemic secular trend. Covid contrast 1 = Cohorts 2016 – 2019 vs. 2020 – 2022. Covid contrast 2 = Cohort 2020 vs. 2021. Covid contrast 3 = Cohort 2021 vs. 2022. Bold = $|z| > 2.0$, linear mixed model random factors: schools (515) and children (98,510), observations = 570,786. For estimates of VCs and CPs see Table S4.

Table S4. Child- and school-related variance components and correlation parameters of the reparameterized LMM for keyage children

	VC	CP						
		Endurance	Coordination	Speed	PowerLOW	PowerUP	Balance	Age
**Child**								
Endurance	0.493	1.00						
Coordination	0.500	0.57	1.00					
Speed	0.515	0.64	0.69	1.00				
PowerLOW	0.564	0.63	0.69	0.81	1.00			
PowerUP	0.448	0.23	0.47	0.44	0.54	1.00		
Balance	0.492	0.21	0.24	0.22	0.28	0.09	1.00	
**School**								
Endurance	0.128	1.00						
Coordination	0.158	0.40	1.00					
Speed	0.113	0.32	0.45	1.00				
PowerLOW	0.074	0.44	0.46	0.46	1.00			
PowerUP	0.061	0.23	0.27	0.33	0.23	1.00		
Balance	0.116	0.00	0.22	0.07	0.17	-0.04	1.00	
Age	0.001	0.74	0.48	0.62	0.60	0.43	0.21	
Sex	0.002	0.15	-0.15	-0.01	0.03	-0.16	0.32	0.51
Pre1 (linear)	0.978							
Pre2 (quadratic)	0.148							
Covid contrast 1	0.032							
Covid contrast 2	0.059							
Covid contrast 3	0.062							

Endurance = cardiorespiratory endurance (i.e., 6-min run test), Coordination = star-run test, Speed = 20-m linear sprint test, PowerLOW = lower limbs muscle power (i.e., standing long jump test), PowerUP = upper limbs muscle power (i.e., ball-push test), balance = static balance (i.e., one-legged-stance test with eyes closed). Cohort contrasts: Covid contrast 1 = Cohorts 2016 – 2019 vs. 2020 – 2022. Covid contrast 2 = Cohort 2020 vs. 2021. Covid contrast 3 = Cohort 2021 vs. cohort 2022. Pre1 (linear) = Linear pre-pandemic secular trend. Pre2 (quadratic) = Quadratic pre-pandemic secular trend. VC = variance component, CP = correlation parameter. VC for Residual = 0.335.

Linear mixed model for keyage children in cohorts 2011 until 2022 with five physical fitness tests

We fit a linear mixed model with data from keyage children in cohorts 2011 to 2022. As the one-legged stance was added to the EMOTIKON test battery in 2016, we only included five physical fitness tests that have been part of the EMOTIKON test battery since 2011. The LMM included linear, quadratic, and cubic pre-pandemic cohort trends. Table S5 shows fixed-effect estimates, standard errors, and z-values of this LMM.

Cardiorespiratory endurance, coordination, and powerUP were lower in the pandemic cohorts 2020 – 2022 compared to the pre-pandemic cohorts 2011 – 2019. For powerLOW, there was no evidence of a significant difference between the average performance in the pre-pandemic and in the pandemic cohorts. For speed, performance was higher in the pandemic cohorts 2020 – 2022 compared to the pre-pandemic cohorts 2011 – 2019. This is different from the negative Covid pandemic effect reported in the results sections in the manuscript. As speed has increased linearly since 2011, the cohort average of 2011 – 2019 is lower than the cohort average of 2016 – 2019. Figure S1 shows the development of third-graders' physical fitness in five physical fitness components from 2011 until 2022.

Table S5. Fixed effect estimates, standard errors and z-values of LMM for keyage children in cohorts 2011 – 2022 and five physical fitness tests.

Source of variance	Fixed-effect estimate	Standard error	z-value
Grand Mean (Intercept)	-0.042	0.009	-4.46
Physical fitness component			
ECS vs. LU	0.019	0.009	**2.02**
EC vs. S	-0.017	0.014	-1.29
E vs. C	0.016	0.017	0.97
L vs. U	0.008	0.013	0.59
Endurance *(6-min run test)*			
Pre1 (linear)	-1.253	0.125	**-10.06**
Pre2 (quadratic)	0.589	0.761	0.77
pre3 (cubic)	1.260	0.454	**2.78**
Covid contrast 1	-0.137	0.010	**-13.71**
Covid contrast 2	-0.021	0.016	-1.32
Covid contrast 3	-0.007	0.017	-0.40
Coordination *(star-run test)*			
pre1 (linear)	-0.365	0.125	**-2.92**
pre2 (quadratic)	-0.429	0.764	-0.56
pre3 (cubic)	0.872	0.456	1.91
Covid contrast 1	-0.163	0.010	**-16.17**
Covid contrast 2	-0.044	0.016	**-2.68**
Covid contrast 3	0.027	0.017	1.55

Source of variance	Fixed-effect estimate	Standard error	z-value
***Speed** (20-m sprint test)*			
Pre1 (linear)	1.199	0.125	**9.60**
Pre2 (quadratic)	0.737	0.764	0.96
Pre3 (cubic)	0.683	0.456	1.50
Covid contrast 1	0.026	0.010	**2.61**
Covid contrast 2	-0.039	0.016	**-2.38**
Covid contrast 3	0.017	0.017	0.98
***PowerLOW** (standing long jump test)*			
Pre1 (linear)	-0.749	0.126	**-5.97**
Pre2 (quadratic)	-1.101	0.770	-1.43
Pre3 (cubic)	1.996	0.459	**4.35**
Covid contrast 1	-0.009	0.010	-0.94
Covid contrast 2	-0.044	0.017	**-2.67**
Covid contrast 3	0.010	0.017	0.56
***PowerUP** (ball-push test)*			
Pre1 (linear)	-0.023	0.123	-0.18
Pre2 (quadratic)	-3.793	0.751	**-5.05**
Pre3 (cubic)	0.717	0.448	1.60
Covid contrast 1	-0.081	0.010	**-8.14**
Covid contrast 2	-0.071	0.016	**-4.43**
Covid contrast 3	0.050	0.017	**2.97**
Age (linear) nested within physical fitness component			
Endurance: a1	0.078	0.009	**8.60**
Coordination: a1	0.287	0.009	**31.19**
Speed: a1	0.222	0.009	**24.15**
PowerLOW: a1	0.215	0.009	**22.97**
PowerUP: a1	0.523	0.009	**59.30**
Sex nested within physical fitness component			
Endurance: Sex	0.458	0.005	**86.73**
Coordination: Sex	0.225	0.005	**42.13**
Speed: Sex	0.302	0.005	**56.55**
PowerLOW: Sex	0.377	0.005	**69.62**
PowerUP: Sex	0.662	0.005	**128.92**

Physical fitness contrasts: ECS vs. LU = endurance, coordination, and speed vs. powerLOW and powerUP. EC vs. S = endurance and coordination vs. speed. E vs. C = endurance vs. coordination. L vs. U = powerLOW vs. powerUP. Cohort contrasts: Pre1 (linear) = Linear pre-pandemic secular trend. Pre2 (quadratic) = Quadratic pre-pandemic secular trend. Pre3 (cubic) = Cubic pre-pandemic secular trend. Covid contrast 1 = Cohorts 2011 – 2019 vs. 2020 – 2022. Covid contrast 2 = Cohort 2020 vs. cohort 2021. Covid contrast 3 = Cohort 2021 vs. 2022. Endurance = cardiorespiratory endurance (i.e., 6-min run test), coordination = star-run test, speed = 20-m linear sprint test, powerLOW = lower limbs muscle power (i.e., standing long jump test), powerUP = upper limbs muscle power (i.e., ball-push test). Bold = $|z| > 2.0$, linear mixed model random factors: schools (532) and children (152,617), observations = 739,699. For estimates of variance components and correlation parameters, see Table S6.

Table S6. Child- and school-related variance components and correlation parameters of the LMM for keyage children in cohorts 2011 – 2022 and five physical fitness tests.

	VC	CP					
		Grand Mean	ECS vs. LU	EC vs. S	E vs. C	L vs. U	Age
**Child**							
Grand Mean (Intercept)	0.339	1.00					
ECS vs. LU	0.243	-0.07	1.00				
EC vs. S	0.292	0.14	0.17	1.00			
E vs. C	0.515	0.10	0.17	0.07	1.00		
L vs. U	0.549	-0.33	0.40	-0.12	0.13	1.00	
**School**							
Grand Mean (Intercept)	0.042	1.00					
ECS vs. LU	0.041	-0.46	1.00				
EC vs. S	0.090	-0.11	0.20	1.00			
E vs. C	0.141	0.13	-0.12	-0.04	1.00		
L vs. U	0.087	-0.23	0.17	0.05	0.03	1.00	
Age	0.004	0.61	-0.30	-0.29	-0.04	-0.20	1.00
Sex	0.002	0.09	-0.03	0.00	-0.23	-0.29	0.19
Pre1 (linear)	4.33						
Pre2 (quadratic)	141.40						
Pre3 (cubic)	53.78						
Covid contrast 1	0.031						
Covid contrast 2	0.060						
Covid contrast 3	0.074						

Physical fitness contrasts: ECS vs. LU = endurance, coordination, and speed vs. powerLOW and powerUP. EC vs. S = endurance and coordination vs. speed. E vs. C = endurance vs. coordination. L vs. U = powerLOW vs. powerUP. Cohort contrasts: Pre1 (linear) = Linear pre-pandemic secular trend. Pre2 (quadratic) = Quadratic pre-pandemic secular trend. Pre3 (cubic) = Cubic pre-pandemic secular trend. Covid contrast 1 = Cohorts 2016 – 2019 vs. 2020 – 2022. Covid contrast 2 = Cohort 2020 vs. 2021. Covid contrast 3 = Cohort 2021 vs. 2022. Endurance = cardiorespiratory endurance (i.e., 6-min run test), Coordination = star-run test, Speed = 20-m linear sprint test, PowerLOW = lower limbs muscle power (i.e., standing long jump test), PowerUP = upper limbs muscle power (i.e., ball-push test), VC = variance component, CP = correlation parameter. VC for Residual = 0.306.

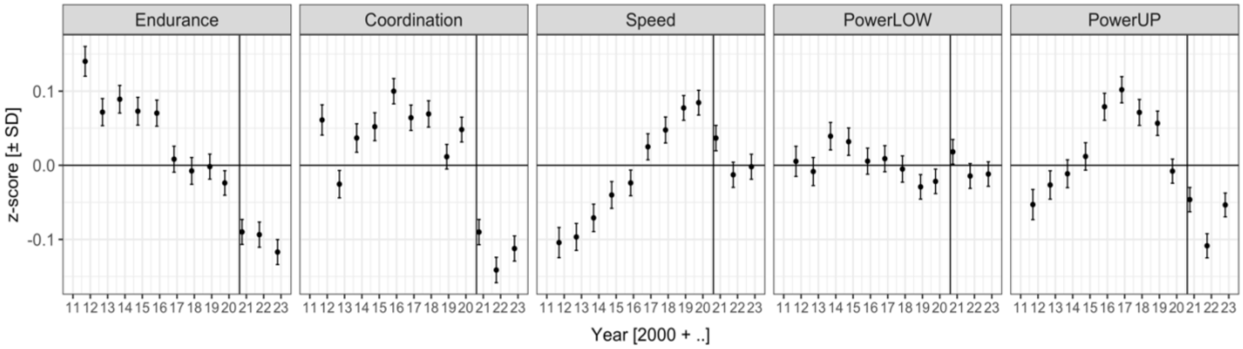

Figure S1. Mean z-scores and 95% CIs for the cohorts 2011 to 2022 for five physical fitness components. Cohort means are based on 739,699 test scores from 152,617 children in 532 schools. Cohort means are shown at the mean test date for each cohort. The vertical line marks the first day of the school year in which the first Covid cohort was tested (August 10, 2020) and separates pre-pandemic cohorts (2011 – 2019) from pandemic cohorts (2020 – 2022). Endurance = cardiorespiratory endurance (i.e., 6-minute-run test), Coordination = star-run test, Speed = 20-m linear sprint test, PowerLOW = lower limbs muscle power (i.e., standing long jump test), PowerUP = upper limbs muscle power (i.e., ball-push test). For coordination and speed, scores were converted from seconds to meter/second (i.e., pace scores; star-run test = 50.912 [m] / time [s]; 20-m linear sprint test = 20 [m] / time [s]).
